# Supplementary material for: A scoping review of medication self-management intervention tools to support persons with traumatic spinal cord injury
Source: PLoS One. 2023 Apr 20;18(4):e0284199. doi: 10.1371/journal.pone.0284199 (PMC10118177; doi:10.1371/journal.pone.0284199)
Supplement: S2 Table — (DOCX) [file pone.0284199.s002.docx]

**S2 Table. Full search strategies for all electronic databases**

1. Ovid MEDLINE: Epub Ahead of Print, In-Process & Other Non-Indexed Citations, Ovid MEDLINE® Daily and Ovid MEDLINE® <1946-Present>

| # | Searches | Results |
| --- | --- | --- |
| 1 | spinal cord compression/ or spinal cord injuries/ or central cord syndrome/ or spinal injuries/ or spinal fractures/ or Spinal Cord Ischemia/ | 76416 |
| 2 | ((spine* or spinal* or vertebr* or lumbar or central cord) adj4 (injur* or contusion* or dysfunct* or laceration* or trauma* or transection* or damag* or fractur* or compress* or weak?n* or atroph* or ischemi* or syndrome* or break* or broke* or dissect* or lesion* or h?emorrhag*)).tw,kf. | 126253 |
| 3 | (fractur* adj3 (burst or chance or compression* or lumber* or sacrum* or thoracic)).tw,kf. | 9320 |
| 4 | (trauma* adj3 myelopath*).tw,kf. | 200 |
| 5 | (hangman* adj2 fractur*).tw,kf. | 344 |
| 6 | stroke/ or brain infarction/ or brain stem infarctions/ or lateral medullary syndrome/ or cerebral infarction/ or infarction, anterior cerebral artery/ or infarction, middle cerebral artery/ or infarction, posterior cerebral artery/ or hemorrhagic stroke/ or ischemic stroke/ or embolic stroke/ or thrombotic stroke/ or stroke, lacunar/ or Stroke Rehabilitation/ | 158256 |
| 7 | (stroke or strokes or poststroke).tw,kf. | 288455 |
| 8 | ((brain* or cerebral* or cerebro* or subcortical or choroidal artery or aca or artery or arteries or lacunar or intracerebral* or cranial* or subdural* or "sub dural" or "sub arachnoid" or subarachnoid or hemispheri*) adj3 (infarct* or h?emorrhag*)).tw,kf. | 96375 |
| 9 | ((cerebrovascular or vascular or brain*) adj3 (arrest* or failure* or insuffician* or attack* or event* or incident* or infarct* or accident*)).tw,kf. | 45566 |
| 10 | transient isch?emic attack*.tw,kf. | 15875 |
| 11 | apoplex*.tw,kf. | 3444 |
| 12 | ((lacunar or cerebral arter* or medullary or vieseaux-wallenberg* or wallenberg*) adj3 syndrome*).tw,kf. | 1508 |
| 13 | or/1-12 | 553997 |
| 14 | prescriptions/ or drug prescriptions/ | 34266 |
| 15 | pharmaceutical preparations/ or drugs, chinese herbal/ or drugs, essential/ or drugs, generic/ or drugs, investigational/ or prescription drugs/ | 131458 |
| 16 | drug therapy/ or drug therapy, combination/ or drug therapy, computer-assisted/ or polypharmacy/ | 211172 |
| 17 | (medicat* or prescri* or drug or drugs or pharmacotherap* or pharmaceutic* or polypharm* or dosing or dose or dosage).tw,kf. | 3364443 |
| 18 | ((Pharmaco* or medicinal) adj3 (treatment* or therap*)).tw,kf. | 67768 |
| 19 | or/14-18 | 3555720 |
| 20 | Self-Management/ or Self Medication/ or Self Administration/ or medication therapy management/ or Self Care/ or Self-Control/ or self concept/ or self-assessment/ or self efficacy/ or Self Report/ or Medication Adherence/ or diagnostic self evaluation/ or self-directed learning as topic/ | 211647 |
| 21 | problem solving/ | 26068 |
| 22 | Decision Making/ | 101776 |
| 23 | Social Interaction/ | 1089 |
| 24 | SELF-HELP GROUPS/ | 9441 |
| 25 | ((self or selve* or onesel* or herself or himself or themsel* or own or home) adj3 (manag* or monitor* or admin* or regulat* or maintain* or direct* or efficac* or care or caring or help* or control* or aid or concept* or assess* or evaluat* or confiden* or esteem* or perception* or treatment* or report* or direct* or tailor* or apprais* or diagnos* or determinat* or reinforc* or training)).tw,kf. | 518932 |
| 26 | ((self or selve* or onesel* or herself or himself or themsel* or own or individual* or home or manag* or adher*) adj3 (medicat* or dose or dosing or prescri* or drug or drugs or pharmacotherap* or pharmaceutic* or polypharm* or admin*)).tw,kf. | 144908 |
| 27 | ((role* or emotion*) adj3 manag*).tw,kf. | 18696 |
| 28 | ((cope or coping) adj3 (behavio?r or skill* or abilit*)).tw,kf. | 9935 |
| 29 | (problem* adj3 solv*).tw,kf. | 60776 |
| 30 | (decision* adj3 (make or making or made or process)).tw,kf. | 214329 |
| 31 | (goal* adj3 (set or sets or setting)).tw,kf. | 9699 |
| 32 | (social adj3 (engag* or interact* or relations* or function*)).tw,kf. | 75849 |
| 33 | Adaptation, Psychological/ | 100771 |
| 34 | (psychologic* adj2 (adapt* or adjust*)).tw,kf. | 5090 |
| 35 | or/20-34 | 1167235 |
| 36 | 13 and 19 and 35 | 4591 |

1. Embase Classic+Embase <1947 to 2022 March 09>

| # | Searches |
| --- | --- |
| 1 | spinal cord injury/ or cervical spinal cord injury/ or spinal cord compression/ or spinal cord transsection/ |
| 2 | spinal cord compression/ |
| 3 | central cord syndrome/ |
| 4 | spine fracture/ or burst fracture/ or cervical spine fracture/ or chance fracture/ or compression fracture/ or lumbar spine fracture/ or sacrum fracture/ or thoracic spine fracture/ or vertebral body fracture/ or vertebral pedicle fracture/ or spine injury/ or cervical spine injury/ or vertebra compression/ or vertebra dislocation/ |
| 5 | spinal cord ischemia/ |
| 6 | ((spine* or spinal* or vertebr* or lumbar or central cord) adj3 (injur* or contusion* or dysfunct* or laceration* or trauma* or transection* or damag* or fractur* or compress* or weak?n* or atroph* or ischemi* or syndrome* or break* or broke* or dissect* or lesion* or h?emorrhag*)).tw,kf. |
| 7 | (fractur* adj2 (burst or chance or compression* or lumber* or sacrum* or thoracic)).tw,kf. |
| 8 | (trauma* adj2 myelopath*).tw,kf. |
| 9 | (hangman* adj2 fractur*).tw,kf. |
| 10 | cerebrovascular accident/ or cardioembolic stroke/ or ischemic stroke/ or lacunar stroke/ |
| 11 | brain infarction/ or brain stem infarction/ |
| 12 | cerebral artery disease/ |
| 13 | ischemic stroke/ or acute ischemic stroke/ or chronic ischemic stroke/ or cryptogenic ischemic stroke/ or subacute ischemic stroke/ or wake up stroke/ or Wallenberg syndrome/ |
| 14 | stroke rehabilitation/ |
| 15 | (stroke or strokes or poststroke).tw,kf. |
| 16 | ((brain* or cerebral* or cerebro* or subcortical or choroidal artery or aca or artery or arteries or lacunar or intracerebral* or cranial* or subdural* or "sub dural" or "sub arachnoid" or subarachnoid or hemispheri*) adj2 (infarct* or h?emorrhag*)).tw,kf. |
| 17 | ((cerebrovascular or vascular or brain*) adj2 (event* or incident* or infarct* or accident* or arrest* or failure* or insuffician* or attack*)).tw,kf. |
| 18 | transient isch?emic attack*.tw,kf. |
| 19 | apoplex*.tw,kf. |
| 20 | ((lacunar or cerebral arter* or medullary or vieseaux-wallenberg* or wallenberg*) adj2 syndrome*).tw,kf. |
| 21 | or/1-20 |
| 22 | drug/ or behind the counter drug/ or chinese drug/ or essential drug/ or generic drug/ or prescription drug/ |
| 23 | prescription/ |
| 24 | drug therapy/ |
| 25 | polypharmacy/ |
| 26 | (medicat* or prescri* or drug or drugs or pharmacotherap* or pharmaceutic* or polypharm*).tw,kf. |
| 27 | ((Pharmaco* or medicinal) adj2 (treatment* or therap*)).tw,kf. |
| 28 | 22 or 23 or 24 or 25 or 26 or 27 |
| 29 | self care/ or self help/ or self medication/ |
| 30 | drug self administration/ |
| 31 | medication therapy management/ |
| 32 | self control/ or self concept/ |
| 33 | self evaluation/ |
| 34 | medication compliance/ |
| 35 | self-directed learning/ |
| 36 | problem solving/ |
| 37 | decision making/ or patient decision making/ |
| 38 | social interaction/ |
| 39 | ((self or selves or onesel* or herself or himself or themsel* or individual* own or home) adj2 (manag* or monitor* or admin* or regulat* or maintain* or direct* or efficac* or care or caring or help* or control* or aid or concept* or assess* or evaluat* or confiden* or esteem* or perception* or treatment* or report* or tailor* or apprais* or diagnos*)).tw,kf. |
| 40 | ((self or selves or onesel* or herself or himself or themsel* or individual* or own or home or manag* or adher*) adj2 (medicat* or dose or dosing or prescri* or drug or drugs or pharmacotherap* or pharmaceutic* or polypharm* or admin*)).tw,kf. |
| 41 | ((role or roles or emotion*) adj2 manag*).tw,kf. |
| 42 | ((cope or coping) adj2 (behavio?r or skill* or abilit*)).tw,kf. |
| 43 | (problem* adj2 solv*).tw,kf. |
| 44 | (decision* adj2 (make or making or made or process)).tw,kf. |
| 45 | ((goal or goals) adj2 (set or sets or setting)).tw,kf. |
| 46 | (social adj2 (enagag* or interact* or relations* or function*)).tw,kf. |
| 47 | psychological adjustment/ |
| 48 | (psychologic* adj2 (adapt* or adjust*)).tw,kf. |
| 49 | or/29-48 |
| 50 | 21 and 28 and 49 |

|  |
| --- |
|  |

1. CINAHL Plus with Full Text (EBSCOhost Research Databases)

| # | Query |
| --- | --- |
| S40 | S14 AND S19 AND S39 |
| S39 | S20 OR S21 OR S22 OR S23 OR S24 OR S25 OR S26 OR S27 OR S28 OR S29 OR S30 OR S31 OR S32 OR S33 OR S34 OR S35 OR S36 OR S37 OR S38 |
| S38 | TI ( (psychologic* N2 (adapt* or adjust*)) ) OR AB ( (psychologic* N2 (adapt* or adjust*)) ) |
| S37 | (MH "Adaptation, Psychological") |
| S36 | TI ( (social N3 (enagag* or interact* or relations* or function*)) ) OR AB ( (social N3 (enagag* or interact* or relations* or function*)) ) |
| S35 | TI ( (goal* N3 (set or sets or setting)) ) OR AB ( (goal* N3 (set or sets or setting)) ) |
| S34 | TI ( (decision* N3 (make or making or made or process)) ) OR AB ( (decision* N3 (make or making or made or process)) ) |
| S33 | TI (problem* N3 solv*) OR AB (problem* N3 solv*) |
| S32 | TI ( ((cope or coping) N3 (behavio?r or skill* or abilit*)) ) OR AB ( ((cope or coping) N3 (behavio?r or skill* or abilit*)) ) |
| S31 | TI ( ((self or selve* or onesel* or herself or himself or themsel* or own or individual* or home or manag* or adher*) N3 (medicat* or dose or dosing or prescri* or drug or drugs or pharmacotherap* or pharmaceutic* or polypharm* or admin*)) ) OR AB ( ((self or selve* or onesel* or herself or himself or themsel* or own or individual* or home or manag* or adher*) N3 (medicat* or dose or dosing or prescri* or drug or drugs or pharmacotherap* or pharmaceutic* or polypharm* or admin*)) ) |
| S30 | TI ( ((role* or emotion*) N3 manag*) ) OR AB ( ((role* or emotion*) N3 manag*) ) |
| S29 | TI ( ((self or selve* or onesel* or herself or himself or themsel* or own or individual* or home) N3 (manag* or monitor* or admin* or regulat* or maintain* or direct* or efficac* or care or caring or help* or control* or aid* or concept* or assess* or evaluat* or confiden* or esteem* or perception* or treatment* or report* or direct* or tailor* or apprais* or diagnos* or determinat* or reinforc* or training)) ) OR AB ( ((self or selve* or onesel* or herself or himself or themsel* or own or individual* or home) N3 (manag* or monitor* or admin* or regulat* or maintain* or direct* or efficac* or care or caring or help* or control* or aid* or concept* or assess* or evaluat* or confiden* or esteem* or perception* or treatment* or report* or direct* or tailor* or apprais* or diagnos* or determinat* or reinforc* or training)) ) |
| S28 | (MH "Decision Making, Patient") OR (MH "Decision Making") |
| S27 | (MH "Problem Solving") |
| S26 | (MH "Self-Diagnosis") |
| S25 | (MH "Self Directed Learning") |
| S24 | (MH "Medication Compliance") |
| S23 | (MH "Self Assessment") |
| S22 | (MH "Self Concept") OR (MH "Self-Efficacy") |
| S21 | (MH "Self Regulation") |
| S20 | (MH "Self-Management") OR (MH "Self Medication") OR (MH "Self-Testing") OR (MH "Self Administration") OR (MH "Self Care") |
| S19 | S15 OR S16 OR S17 OR S18 |
| S18 | TI ( (Pharmaco* or medicinal) N3 (treatment* or therap*) ) OR AB ( (Pharmaco* or medicinal) N3 (treatment* or therap*) ) |
| S17 | TI ( (medicat* or prescri* or drug or drugs or pharmacotherap* or pharmaceutic* or polypharm* or dosing or dose or dosage) ) OR AB ( (medicat* or prescri* or drug or drugs or pharmacotherap* or pharmaceutic* or polypharm* or dosing or dose or dosage) ) |
| S16 | (MH "Drug Therapy") OR (MH "Drug Therapy, Combination") OR (MH "Drug Therapy, Computer Assisted") OR (MH "Polypharmacy") OR (MH "Prescriptions, Drug") |
| S15 | (MH "Drugs") OR (MH "Drugs, Prescription") OR (MH "Drugs, Generic") |
| S14 | S1 OR S2 OR S3 OR S4 OR S5 OR S6 OR S7 OR S8 OR S9 OR S10 OR S11 OR S12 OR S13 |
| S13 | TI ( ((lacunar or cerebral arter* or medullary or vieseaux-wallenberg* or wallenberg*) N3 syndrome*) ) OR AB ( ((lacunar or cerebral arter* or medullary or vieseaux-wallenberg* or wallenberg*) N3 syndrome*) ) |
| S12 | TI apoplex* OR AB apoplex* |
| S11 | TI transient isch?emic attack* OR AB transient isch?emic attack* |
| S10 | TI ( ((cerebrovascular or vascular or brain*) N3 (event* or incident* or infarct* or accident* or arrest* or failure* or insuffician* or attack* )) ) OR AB ( ((cerebrovascular or vascular or brain*) N3 (event* or incident* or infarct* or accident* or arrest* or failure* or insuffician* or attack* )) ) |
| S9 | TI ( ((brain* or cerebral* or cerebro* or subcortical or choroidal artery or aca or artery or arteries or lacunar or intracerebral* or cranial* or subdural* or "sub dural" or "sub arachnoid" or subarachnoid or hemispheri*) N3 (infarct* or h?emorrhag*)) ) OR AB ( ((brain* or cerebral* or cerebro* or subcortical or choroidal artery or aca or artery or arteries or lacunar or intracerebral* or cranial* or subdural* or "sub dural" or "sub arachnoid" or subarachnoid or hemispheri*) N3 (infarct* or h?emorrhag*)) ) |
| S8 | TI ( (stroke or strokes or poststroke) ) OR AB ( (stroke or strokes or poststroke) ) |
| S7 | (MH "Stroke Patients") |
| S6 | (MH "Stroke") OR (MH "Hemorrhagic Stroke") OR (MH "Cerebral Infarction") OR (MH "Ischemic Stroke") OR (MH "Embolic Stroke") OR (MH "Stroke, Lacunar") |
| S5 | TI (hangman* N2 fractur*) OR AB (hangman* N2 fractur*) |
| S4 | TI (trauma* N3 myelopath*) OR AB (trauma* N3 myelopath*) |
| S3 | TI ( (fractur* N3 (burst or chance or compression* or lumber* or sacrum* or thoracic)) ) OR AB ( (fractur* N3 (burst or chance or compression* or lumber* or sacrum* or thoracic)) ) |
| S2 | TI ( ((spine* or spinal* or vertebr* or lumbar or central cord) N4 (injur* or contusion* or dysfunct* or laceration* or trauma* or transection* or damag* or fractur* or compress* or weak?n* or atroph* or ischemi* or syndrome* or break* or broke* or dissect* or lesion* or h?emorrhag*)) ) OR AB ( ((spine* or spinal* or vertebr* or lumbar or central cord) N4 (injur* or contusion* or dysfunct* or laceration* or trauma* or transection* or damag* or fractur* or compress* or weak?n* or atroph* or ischemi* or syndrome* or break* or broke* or dissect* or lesion* or h?emorrhag*)) ) |
| S1 | (MH "Spinal Cord Compression") OR (MH "Spinal Cord Injuries") OR (MH "Central Cord Syndrome") OR (MH "Spinal Injuries") OR (MH "Spinal Fractures") OR (MH "Fractures, Vertebral Compression") |

1. APA PsycInfo <1806 to March Week 1 2022>

| # | Searches |
| --- | --- |
| 1 | spinal cord injuries/ |
| 2 | ((spine* or spinal* or vertebr* or lumbar or central cord) adj4 (injur* or contusion* or dysfunct* or laceration* or trauma* or transection* or damag* or fractur* or compress* or weak?n* or atroph* or ischemi* or syndrome* or break* or broke* or dissect* or lesion* or h?emorrhag*)).ti,ab. |
| 3 | (fractur* adj3 (burst or chance or compression* or lumber* or sacrum* or thoracic)).ti,ab. |
| 4 | (trauma* adj3 myelopath*).ti,ab. |
| 5 | (hangman* adj10 fractur*).ti,ab. |
| 6 | cerebrovascular accidents/ or cerebral ischemia/ or cerebral hemorrhage/ or thromboses/ or embolisms/ or neurorehabilitation/ |
| 7 | (stroke or strokes or poststroke).ti,ab. |
| 8 | ((brain* or cerebral* or cerebro* or subcortical or choroidal artery or aca or artery or arteries or lacunar or intracerebral* or cranial* or subdural* or "sub dural" or "sub arachnoid" or subarachnoid or hemispheri*) adj3 (infarct* or h?emorrhag*)).ti,ab. |
| 9 | ((cerebrovascular or vascular or brain*) adj3 (event* or incident* or infarct* or accident* or arrest* or failure* or insuffician* or attack*)).ti,ab. |
| 10 | transient isch?emic attack*.ti,ab. |
| 11 | apoplex*.ti,ab. |
| 12 | ((lacunar or cerebral arter* or medullary or vieseaux-wallenberg* or wallenberg*) adj3 syndrome*).ti,ab. |
| 13 | or/1-12 [traumatic spinal cord injury + stroke] |
| 14 | prescription drugs/ or drug therapy/ or drugs/ or "prescribing (drugs)"/ |
| 15 | "medicinal herbs and plants"/ or generic drugs/ or nonprescription drugs/ |
| 16 | polypharmacy/ |
| 17 | (medicat* or prescri* or drug or drugs or pharmacotherap* or pharmaceutic* or polypharm* or dosing or dose or dosage).ti,ab. |
| 18 | ((Pharmaco* or medicinal) adj3 (treatment* or therap*)).ti,ab. |
| 19 | or/14-18 [medication] |
| 20 | self-management/ or self-medication/ or drug self administration/ or self-care/ or self-control/ or self-evaluation/ or self-efficacy/ or self-report/ or treatment compliance/ |
| 21 | problem solving/ |
| 22 | decision making/ |
| 23 | social interaction/ |
| 24 | coping behavior/ or goal setting/ |
| 25 | self-help techniques/ or support groups/ or self-monitoring/ or self-referral/ or self-regulation/ or self-instructional training/ or self-determination/ or self-reinforcement/ |
| 26 | ((self or selve* or onesel* or herself or himself or themsel* or own or home) adj3 (manag* or monitor* or admin* or regulat* or maintain* or direct* or efficac* or care or caring or help* or control* or aid or concept* or assess* or evaluat* or confiden* or esteem* or perception* or treatment* or report* or direct* or tailor* or apprais* or diagnos* or determinat* or reinforc* or training)).ti,ab. |
| 27 | ((self or selve* or onesel* or herself or himself or themsel* or own or home or manag* or adher*) adj3 (medicat* or dose or dosing or prescri* or drug or drugs or pharmacotherap* or pharmaceutic* or polypharm* or admin*)).ti,ab. |
| 28 | ((role* or emotion*) adj3 manag*).ti,ab. |
| 29 | ((cope or coping) adj3 (behavio?r or skill* or abilit*)).ti,ab. |
| 30 | (problem* adj3 solv*).ti,ab. |
| 31 | (decision* adj3 (make or making or made or process)).ti,ab. |
| 32 | (goal* adj3 (set or sets or setting)).ti,ab. |
| 33 | (social adj3 (engag* or interact* or relations* or function*)).ti,ab. |
| 34 | emotional adjustment/ |
| 35 | (psychologic* adj2 (adapt* or adjust*)).ti,ab. |
| 36 | or/20-34 [self-management] |
| 37 | 13 and 19 and 36 |

1. Clarivate Web of Science Core Collection
2. TS=((spine* or spinal* or vertebr* or lumbar) NEAR/2 (injur* or contusion* or dysfunct* or laceration* or trauma* or transection* or damag* or fractur* or compress* or weak* or atroph* or ischemi* or syndrome* or break* or broke* or dissect* or lesion* or hemorrhag*))
3. TS=(fractur* NEAR/2 (burst or chance or compression* or lumber* or sacrum* or thoracic))
4. TS=(trauma* NEAR/2 myelopath*)
5. TS=(hangman* NEAR/2 fractur*)
6. TS=(stroke or strokes or poststroke)
7. TS=((brain* or cerebral* or cerebro* or subcortical or aca or artery or arteries or lacunar or intracerebral* or cranial* or subdural* or subarachnoid or hemispheri*) NEAR/2 (infarct* or hemorrhag*))
8. TS=((cerebrovascular or vascular or brain*) NEAR/2 (event* or incident* or infarct* or accident* or arrest* or failure* or insuffician* or attack*))
9. TS=transient isch?emic attack*
10. TS=apoplex*
11. TS=((lacunar or medullary or vieseaux-wallenberg* or wallenberg*) NEAR/2 syndrome*)
12. #10 OR #9 OR #8 OR #7 OR #6 OR #5 OR #4 OR #3 OR #2 OR #1
13. TS=(medicat* or prescri* or drug or drugs or pharmacotherap* or pharmaceutic* or polypharm* or dosing or dose or dosage)
14. TS=((Pharmaco* or medicinal) NEAR/2 (treatment* or therap*))
15. #12 OR #13
16. TS=((self or selve* or onesel* or herself or himself or themsel* or own or individual* or home) NEAR/2 (manag* or monitor* or admin* or regulat* or maintain* or direct* or efficac* or care or caring or help* or control* or aid* or concept* or assess* or evaluat* or confiden* or esteem* or perception* or treatment* or report* or direct* or tailor* or apprais* or diagnos*))
17. TS=((self or selve* or onesel* or herself or himself or themsel* or own or individual* or home or manag* or adher*) NEAR/2 (medicat* or dose or dosing or prescri* or drug or drugs or pharmacotherap* or pharmaceutic* or polypharm* or admin*))
18. TS=((role* or emotion*) NEAR/2 manag*)
19. TS=((cope or coping) NEAR/2 (behavio?r or skill* or abilit*))
20. TS=(problem* NEAR/2 solv*)
21. TS=(decision* NEAR/2 (make or making or made or process))
22. TS=(goal* NEAR/2 (set or sets or setting))
23. TS=(social NEAR/2 (engag* or interact* or relations* or function*))
24. TS=(psychologic* NEAR/2 (adapt* or adjust*))
25. #15 OR #16 OR #17 OR #18 OR #19 OR #20 OR #21 OR #22 OR #23
26. #11 AND #14 AND #24
