# Supplementary material for: A scoping review of medication self-management intervention tools to support persons with traumatic spinal cord injury
Source: PLoS One. 2023 Apr 20;18(4):e0284199. doi: 10.1371/journal.pone.0284199 (PMC10118177; doi:10.1371/journal.pone.0284199)
Supplement: Attachment — Submitted filename: SCI MSM ScR_Response to Review_20230207.docx [file pone.0284199.s003.docx]

February 7, 2023

Dr. Emily Chenette,

Editor-in-Chief

PLOS ONE

**RE: Response to Reviews**

PONE-D-22-28883

A scoping review of medication self-management interventions for persons with traumatic spinal cord injury

PLOS ONE

Dear Emily Chenette,

Thank you for considering our manuscript to be published in PLOS ONE. We appreciate the reviewers’ comments and have outlined a detailed response to each of the comments.

| Journal Requirements | |
| --- | --- |
| When submitting your revision, we need you to address these additional requirements.  1. Please ensure that your manuscript meets PLOS ONE's style requirements, including those for file naming. The PLOS ONE style templates can be found at <https://journals.plos.org/plosone/s/file?id=wjVg/PLOSOne_formatting_sample_main_body.pdf> and  <https://journals.plos.org/plosone/s/file?id=ba62/PLOSOne_formatting_sample_title_authors_affiliations.pdf>  Please review your reference list to ensure that it is complete and correct. If you have cited papers that have been retracted, please include the rationale for doing so in the manuscript text, or remove these references and replace them with relevant current references. Any changes to the reference list should be mentioned in the rebuttal letter that accompanies your revised manuscript. If you need to cite a retracted article, indicate the article’s retracted status in the References list and also include a citation and full reference for the retraction notice. | We have confirmed that the manuscript meets PLOS ONE’s style requirements and naming conventions.  We have also reviewed the reference list to ensure it is complete and correct. No changes were made. |
| Reviewer #1 | |
| Reviewer #1: Thank you for shedding light on this topic. There is a dearth of literation integrating the patient and caregiver voices, with the exception of some collaborative care model data. This paper has a remarkable potential to bring this issue into the mainstream through PLOS, but I suggest a minor revision. | Thank you for this comment and for your recommendations. |
| Line 220-222 Consecutive sentences “All of the articles reported the sex of the participants, and all included a higher proportion of male participants than female [32-34]. None reported the participants’ gender.” may be misconstrued to be internally inconsistent. Consider re-writing for text flow. | We have revised this section to improve flow. |
| Overall, the findings of this scoping review paper would be valuable to the discipline of collaborative care, hospice, and Bio-ethics, advocating for enhanced patient autonomy. | Thank you for this comment. |
| Reviewer #2 | |
| Reviewer #2: The article is an interesting read. Assimilating the available articles and comparing the three literatures that offer tools for self-management of medication was done tactfully, carefully separating the outcomes- per behavioral outcomes, learning outcomes and clinical outcomes were thoughtful. | Thank you for this comment. |
| The only recommendation would be modifying the title: The scoping review of medication self-management, provided an initial thought to me- if patients with SCI struggled with self-medication challenges, only to realize after reading the article that they struggle managing their polypharmacy/medications independently without appropriate tools. One recommendation for the title could be- Scoping Review of Self-Management Intervention tools utilized for treatment of persons with Traumatic Spinal Cord Injury. | Thank you for this suggestion – we have revised the title. |
| Reviewer #3 | |
| Reviewer #3: The manuscript by Cadel et al. studies the important topic of self-management interventions in spinal cord injury (SCI) considering the chronicity of the condition, the short- and long-term impact on general health, and the quality of life of the patient and their caregivers. | Thank you. |
| Strengths of the paper: - The abstract describes the findings of the study as well as suggestions on what can be done.  - The introduction to the article is well organized.  - Methods and results are clearly described.  - The discussion and conclusion further elaborate on the challenges in the treatment of patients with SCI and strategies for mitigating these challenges. | Thank you for these comments. |
| Some recommendations: - As the authors have pointed out, self-management is not consistently defined. It may be worth adding a brief commentary on the findings of other publications to develop a model for self-management for SCI, for example, by Munce et al. (Munce, S.E.P., Webster, F., Fehlings, M.G. et al. Meaning of self-management from the perspective of individuals with traumatic spinal cord injury, their caregivers, and acute care and rehabilitation managers: an opportunity for improved care delivery. BMC Neurol 16, 11). | Thank you for this recommendation, we have included some commentary from the work by Munce and colleagues into the discussion. |
